# Diagnostic performance of donor-derived cell-free DNA for acute rejection in kidney allograft biopsies

**DOI:** 10.1590/2175-8239-JBN-2025-0285en

**Published:** 2026-03-30

**Authors:** Mônica Rika Nakamura, Renato Demarchi Foresto, Vitoria Regina da Silva Gomes, Henrique Machado Proença, Lúcio Requião-Moura, José Medina-Pestana, Helio Tedesco-Silva

**Affiliations:** 1 Fundação Oswaldo Ramos, Hospital do Rim, São Paulo, SP, Brazil.; 2 Universidade Federal de São Paulo, Departamento de Medicina, Disciplina de Nefrologia, São Paulo, SP, Brazil.

**Keywords:** Kidney Transplantation, Graft Rejection, Donor-Derived Cell-Free DNA, Kidney Biopsy, Noninvasive Biomarker, Diagnosis

## Abstract

**Objective:**

To determine the performance of donor-derived cell-free DNA (dd-cfDNA) as a noninvasive biomarker for the diagnosis of acute rejection in for-cause kidney transplant biopsies.

**Methods:**

This cross-sectional single-center study (between May 2021 and June 2022) included for-cause biopsies performed in kidney transplant recipients with acute graft dysfunction (AGD) or suboptimal graft function (SGF). dd-cfDNA levels were correlated with histological diagnosis according to the Banff 2022 classification.

**Results:**

Among 492 biopsies, 80.7% were performed for AGD and 19.3% for SGF. The distribution of histological phenotypes was 10.2% (category 1), 6.5% (category 2), 6.9% (category 3), 7.9% (category 4), 33.3% (category 5), and 35.2% (category 6). The respective median dd-cfDNA values were 0.25% (IQR 0.16–0.46), 1.88% (IQR 0.92–5.11), 0.45% (IQR 0.26–0.63), 0.51% (IQR 0.34–1.11), 0.27% (IQR 0.15–0.45), and 0.38% (IQR 0.23–0.64). Category 2 presented a higher median dd-cfDNA compared with the other groups (p < 0.001). The area under the curve (AUC) was 0.77 for acute rejection (categories 2 and 4), with a sensitivity of 50.7%, a specificity of 91.2%, a positive predictive value of 49.3%, a negative predictive value of 91.6%, and an accuracy of 85.4%. Similar results were observed in biopsies for AGD or SGF. The dd-cfDNA with the highest diagnostic performance for acute rejection was 0.81%, with optimal thresholds of 0.46% for AGD and 0.81% for SGF biopsies.

**Conclusion:**

In this cohort, dd-cfDNA showed moderate diagnostic performance for acute graft rejection and high negative predictive value. dd-cfDNA threshold diagnostic varied according to the type of for-cause biopsies (AGD or SGF).

## Introduction

Acute graft dysfunction is one of the main challenges in kidney transplantation, with acute rejection being a determining factor for long-term graft loss^1,2^. Studies indicate that the incidence of acute rejection ranges from 6% to 16% in the first year post-transplant, depending on the clinical context and the therapeutic strategies adopted^3,4,5,6^.

Suspicion of acute rejection often arises with an increase in serum creatinine, a widely used biomarker due to its accessibility and low cost^7^. However, its elevation may be delayed and influenced by extrarenal factors such as age, muscle mass, and patient hydration status^7^. Furthermore, variations in creatinine levels do not specify the cause of graft dysfunction, making diagnostic confirmation through kidney graft biopsy essential in clinical practice.

Kidney biopsy is considered the gold standard for diagnosing graft rejection, allowing histopathological analysis of lesions and differentiation between cellular and humoral rejection^5^. However, it is an invasive procedure associated with complications such as macroscopic hematuria, perirenal hematoma, and, in severe cases, the need for surgical intervention^8,9,10^. Thus, there is a need for less invasive methods that allow earlier and more accurate detection of rejection than changes in serum creatinine, while complementing, rather than replacing, kidney biopsy as the diagnostic gold standard.

In this context, several biomarkers have been studied to provide early and noninvasive diagnosis of kidney graft rejection^11,12^. Donor-derived cell-free DNA (*dd-cfDNA*) has emerged as a promising tool for monitoring rejection, enabling the detection of rejection episodes by quantifying DNA released from graft cells into the recipient’s bloodstream^13^. Evidence suggests that increased levels of *dd-cfDNA* are directly correlated with rejection events, supporting its potential as an alternative or complement to kidney biopsy^14^.

The dd-cfDNA offers several advantages, including early detection of rejection and its noninvasive nature. However, its clinical use still faces challenges, such as high costs and the need for standardized cutoff values to optimize its clinical application^15,16,17,18,19^. Nonetheless, the incorporation of dd-cfDNA as a biomarker in kidney transplantation represents a significant advance, with the potential to reduce the need for unnecessary kidney graft biopsies and to contribute to improving long-term graft outcomes by enabling earlier and more accurate detection and treatment of alloimmune injury.

Despite growing international evidence, data on the diagnostic performance of dd-cfDNA in Brazilian kidney transplant recipients (KTRs) are lacking. Therefore, this study aimed to evaluate the diagnostic performance of the Prospera dd-cfDNA assay as a noninvasive biomarker for biopsy-proven acute rejection in for-cause kidney allograft biopsies in a Brazilian cohort.

## Methods

This is a coss-sectional, exploratory, non interventional, single-center study designed to evaluate the performance of the Prospera dd-cfDNA test as a diagnostic tool for acute rejection in KTRs. Between May 17, 2021, and June 6, 2022, we screened all consecutive adult KTRs who underwent a for-cause kidney allograft biopsy.

Eligible patients included adults aged 18 years or older, recipients of living- or deceased-donor kidneys, who were scheduled for a for-cause allograft biopsy and agreed to participate by signing the informed consent form. Patients were excluded if the biopsy was performed within the first 15 days after transplantation, if they were recipients of an HLA-identical living-donor kidney, if they had previously received another solid organ transplant, or if they were pregnant. Once the patients met the inclusion and exclusion criteria, they were invited to participate in the study and signed an informed consent form.

Biopsies were indicated either for acute graft dysfunction (AGD, defined as a rise in serum creatinine compatible with possible acute rejection or other acute graft injury) or for suboptimal graft function (SGF, defined as incomplete recovery of graft function in the early post-transplant period, not referring to incomplete recovery following later episodes of acute graft injury), according to institutional criteria. Graft biopsy results were analyzed by one experienced renal pathologist from our institution and classified according to the Banff 2022 Classification^20^. Banff category 1 comprised biopsies with normal histology or only nonspecific/minimal changes. Category 2 corresponded to antibody-mediated rejection. Category 3 included borderline changes suggestive, but not diagnostic, of T-cell–mediated rejection. Category 4 encompassed T-cell–mediated rejection. Category 5 comprised chronic or chronic active rejection phenotypes, and category 6 included other diagnoses not related to rejection (such as recurrent or *de novo* glomerular diseases, interstitial fibrosis and tubular atrophy, or non-alloimmune lesions). For the diagnostic performance analyses, biopsies fulfilling Banff category 2 or category 4 were considered to show biopsy-proven acute rejection, whereas biopsies classified in categories 1, 3, 5, and 6 were grouped as nonrejection phenotypes.

Subsequently, for dd-cfDNA assessment, two 10 mL peripheral blood samples were collected in EDTA tubes immediately before the biopsy procedure. Plasma samples were handled according to the manufacturer’s instructions, centrifuged, stored at –80°C, and subsequently shipped under controlled temperature to the Natera laboratory (San Carlos, CA, USA) for analysis using the Prospera assay. dd-cfDNA levels were quantified using targeted next-generation sequencing and expressed as the percentage of donor-derived fragments relative to total cell-free DNA in plasma. For the primary analysis, and in line with previous studies and the Prospera assay labeling, a dd-cfDNA value ≥ 1.0% was prespecified as a positive test result for the detection of biopsy-proven acute rejection. The test results were not used to guide clinical decisions and did not interfere with medical management.

Clinical and laboratory data were extracted from the institutional electronic medical records using a standardized case report form. Continuous variables were presented as medians and interquartile ranges (IQR), and categorical variables as absolute numbers and percentages. The distribution of dd-cfDNA values across Banff histological categories was explored using nonparametric tests for independent samples. In the first step, we evaluated the diagnostic performance of the prespecified dd-cfDNA threshold of 1.0% for the detection of biopsy-proven acute rejection by calculating sensitivity, specificity, positive predictive value, negative predictive value, and overall accuracy, with corresponding 95% confidence intervals. In the second step, receiver operating characteristic (ROC) curves were constructed, and the area under the curve (AUC) was estimated. Optimal dd-cfDNA cutoff points for the overall cohort and for the subgroups of biopsies performed for acute graft dysfunction and for suboptimal graft function were determined using the Youden Index, without fixing the 1.0% threshold *a priori*. All statistical analyses were performed using the Statistical Package for the Social Sciences (SPSS), version 29 (IBM Corp., Released 2022)^21^. A p-value < 0.05 was considered statistically significant, with a 95% confidence interval. Clinical data were extracted from electronic medical records in the institution’s system and entered into the REDCap platform, with subsequent export for statistical analysis. The study protocol, as well as the informed consent form, was submitted to and approved by the Research Ethics Committee (approval number: 6,566,447). All study procedures were performed only after ethics committee approval.

## Results

**Figure 1:**
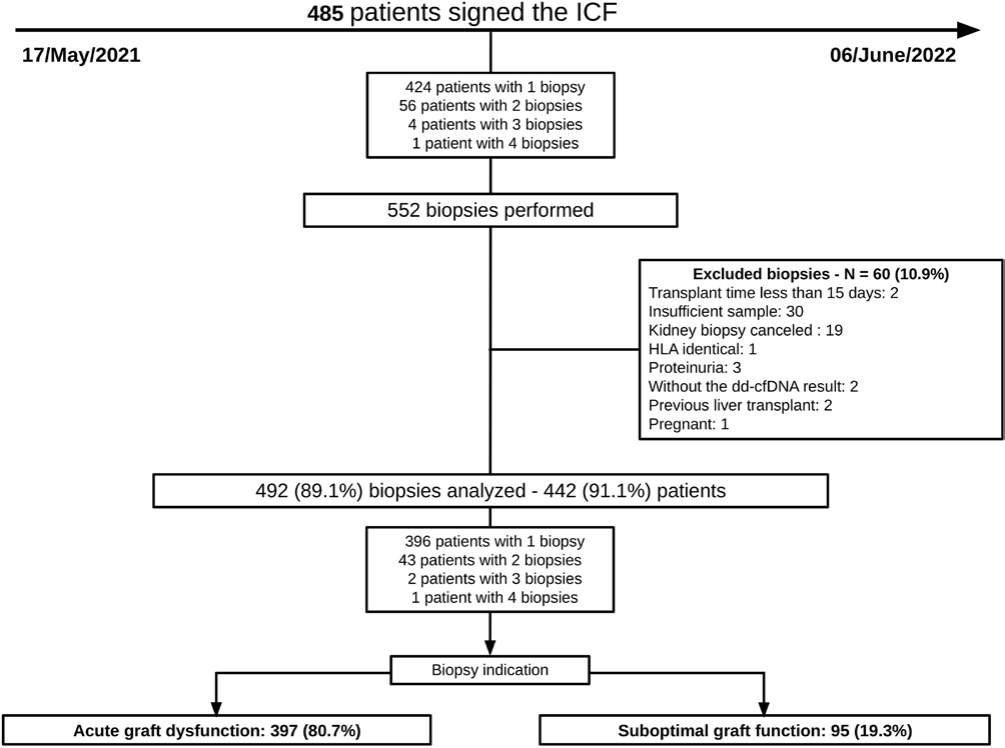
Disposition of study samples.

The study population consisted of a total of 492 biopsies from 446 KTRs, predominantly male (65%), Caucasian (63.4%), with a median age of 44 years (IQR: 34–55 years). Among recipients, 48.6% had chronic kidney disease of indeterminate etiology. Furthermore, 95.1% were recipients of a first kidney transplant, and 96.1% had undergone hemodialysis. Regarding donors, the median age was 48 years (IQR: 38–57 years), 53.7% were male, and 54.3% were Caucasian. There was a predominance of deceased donors (77.2%), with 23.9% having a Kidney Donor Profile Index (KDPI) > 85%. Regarding transplant characteristics, any positivity for panel reactive antibodies (PRA) was present in 23.2% of patients for class I and in 8.7% for class II. The median ABDR mismatch was 2.5. The mean cold ischemia time was 19.0 hours, and 141 patients (28.7%) had delayed graft function (DGF). The main indication for graft biopsy was acute graft dysfunction (AGD), identified in 80.7% of cases. The remaining 19.3% of patients underwent the procedure due to suboptimal graft function (SGF). The median time from transplant to biopsy was 17.5 months, with an IQR of 3.0 to 52.2 months (Figure 1, Table 1).

**Table 1:** Demographics of the study population

Variable	Total (N = 492)	AGD (N = 397)	SGF (N = 95)
Recipient age, years (IQR)	44 (34.0–55.0)	45 (34.0–55.0)	43 (34.5–55.0)
Recipient sex, male, N (%)	318 (65)	244 (61.5)	53 (64)
Recipient ethnicity, N (%)			
White	312 (63.4)	258 (65.0)	54 (56.8)
Black/mixed	179 (36.4)	138 (34.8)	41 (43.2)
Other	1 (0.2)	1 (0.3)	0 (0)
Cause of chronic kidney disease, N (%)			
Glomerulonephritis	94 (19.0)	81 (20.4)	13 (13.7)
Diabetes mellitus	51 (10.4)	44 (11.1)	7 (7.4)
Unknown	239 (48.6)	193 (48.6)	46 (48.4)
Other	108 (22.0)	79 (19.9)	29 (30.5)
Dialysis vintage, months (IQR)	30 (16.0–59.0)	30 (16.0–60.0)	30 (16.0–58.0)
Dialysis modality, hemodialysis, N (%)	473 (96.1)	380 (95.7)	93 (97.9)
Retransplantation, N (%)	24 (4.9)	19 (4.8)	5 (5.3)
Donor age, years (IQR)	48 (38.0–57.0)	47 (38.0–56.0)	52 (42.0–58.5)
Donor sex, male, N (%)	264 (53.7)	211 (53.1)	53 (55.8)
Donor ethnicity, N (%)			
White	267 (54.3)	217 (54.7)	50 (52.6)
Black/mixed	221 (44.9)	176 (44.3)	45 (47.4)
Other	4 (0.8)	4 (1.0)	0 (0)
Donor type, deceased, N (%)	380 (77.2)	296 (74.6)	84 (88.4)
Deceased donors			
KDPI, % (IQR)	65 (44.0–84.3)	64 (44.0–82.0)	67 (48.0–88.0)
KDPI > 85%, N (%)	91 (23.9)	67 (23.0)	24 (29.0)
PRA Class I > 0%, N (%)	114 (23.2)	98 (24.7)	16 (16.8)
PRA Class II > 0%, N (%)	43 (8.7)	39 (9.8)	4 (4.2)
Cold ischemia time, hours (IQR)	19.0 (10.0–25.0)	19.0 (2.0–24.0)	23 (15.0–30.0)
Delayed graft function, N (%)	141 (28.7)	107 (27.0)	34 (35.8)
Induction, anti-thymocyte globulin, N (%)	491 (99.8)	397 (100)	94 (98.9)
Immunosuppression, N (%)			
Tacrolimus + Mycophenolate	256 (52.0)	200 (50.4)	56 (58.9)
Tacrolimus + Azathioprine	152 (30.9)	123 (31.0)	29 (30.5)
Tacrolimus + mTOR inhibitor	68 (13.8)	59 (14.9)	9 (9.5)
Other	16 (3.3)	15 (3.8)	1 (1.1)
Time post-transplant, months (IQR)	17.5 (3.0–52.2)	31 (8.2–63.2)	1.4 (1.2–2.0)
Post-biopsy treatment, yes, N (%)	106 (21.5)	92 (22.9)	14 (14.7)
Methylprednisolone	90 (84.9)	78 (84.8)	12 (85.7)
Anti-thymocyte globulin	7 (6.6)	5 (5.4)	2 (14.3)
Immunoglobulin	3 (2.8)	3 (3.3)	0 (0)
Methylprednisolone + immunoglobulin	4 (3.8)	4 (4.3)	0 (0)
Antibiotic	2 (1.9)	2 (2.2)	0 (0)

Abbreviations – N: Number; AGD: Acute Graft Dysfunction; SGF: Suboptimal Graft Function; IQR: Interquartile Range; KDPI: Kidney Donor Profile Index; PRA: Panel Reactive Antibody; HLA: Human Leukocyte Antigen; mTOR: mammalian target of rapamycin inhibitor. Note – Values are expressed as median (1st IQR; 3rd IQR) and absolute values (%).

A total of 492 dd-cfDNA tests were performed, with results showing a mean value of 0.81%, ranging from 0.01% to 21.02%. The median was 0.35%, with an IQR of 0.18% to 0.63%. Most of the study population, corresponding to 419 cases (85.2%), had dd-cfDNA values below 1%.

**Figure 2:**
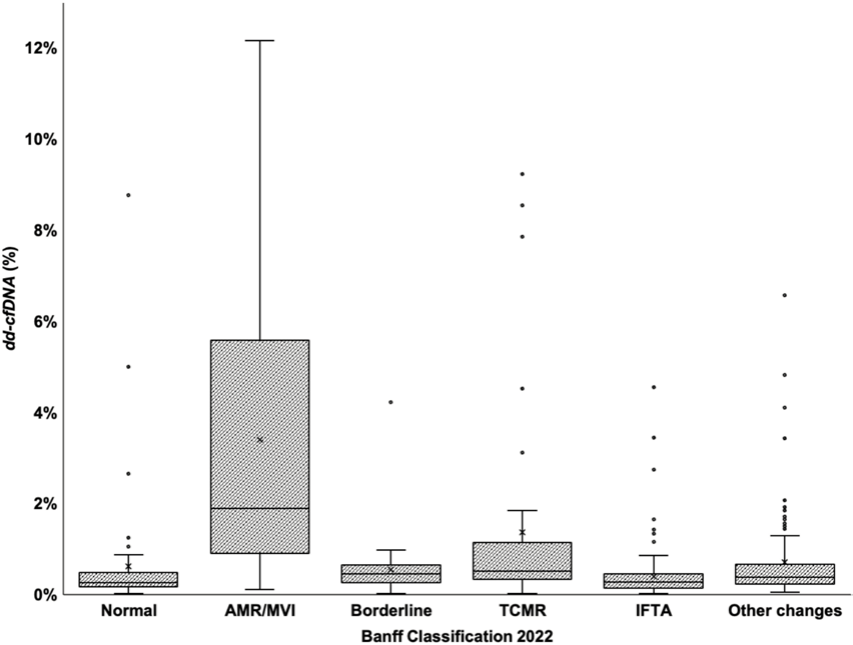
Boxplot showing dd-cfDNA test and 2022 Banff Category.

Figure 2, represented by a boxplot of dd-cfDNA percentages according to the Banff 2022 classification, shows greater dispersion and higher values in category 2 compared with the other categories (p < 0.001). Biopsy-proven acute rejection was identified in 71 biopsies, corresponding to 14.4% of all for-cause biopsies in the cohort. Among these, 32 biopsies were classified as antibody-mediated rejection (Banff category 2) and 39 as T-cell-mediated rejection (Banff category 4). The distribution of histological phenotypes was 10.2% (N = 50) in category 1, 6.5% (N = 32) in category 2, 6.9% (N = 34) in category 3, 7.9% (N = 39) in category 4, 33.3% (N = 164) in category 5, and 35.2% (N = 173) in category 6. The respective median dd-cfDNA percentages were 0.25% (IQR 0.16–0.46), 1.88% (IQR 0.92–5.11), 0.45% (IQR 0.26–0.63), 0.51% (IQR 0.34–1.11), 0.27% (IQR 0.15–0.45), and 0.38% (IQR 0.23–0.64).

**Figure 3:**
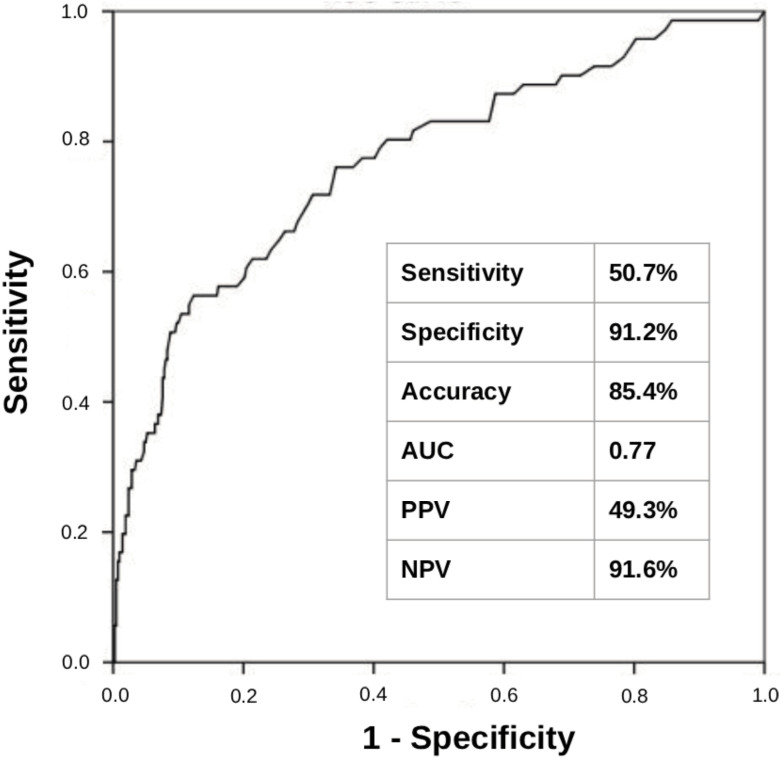
Analysis of dd-cfDNA test performance in the total population.

Using the prespecified threshold of 1.0% to define a positive dd-cfDNA test, the assay showed a sensitivity of 50.7% and a specificity of 91.2% for the detection of biopsy-proven acute rejection, with an overall accuracy of 85.4%. The area under the curve (AUC) was 0.77, suggesting a good ability of the test to discriminate between patients with and without rejection. The positive predictive value (PPV) was 49.3%, and the negative predictive value (NPV) reached 91.6% (Figure 3).

The optimal cutoff point for the dd-cfDNA test to diagnose rejection was determined for the overall cohort (N = 492) based on the Youden Index. The analysis revealed a cutoff of 0.81%, with a sensitivity of 56.3%, a specificity of 87.6%, and an accuracy of 83.1% for differentiating between active rejection and absence of rejection. The positive predictive value was 43.5%, and the negative predictive value was 92.3%. Overall, 81.3% of the samples had dd-cfDNA levels below the established cutoff point.

Furthermore, the optimal dd-cfDNA cutoff point was determined for the group that underwent biopsy due to acute graft dysfunction (N = 397). The optimal cutoff for dd-cfDNA was 0.46%, with a sensitivity of 75.4%, a specificity of 68.2%, and an accuracy of 69.3% for differentiating between active rejection and absence of rejection. The positive predictive value was 30.1%, and the negative predictive value was 93.9%. In this population, 61.5% of the samples had dd-cfDNA levels below the established cutoff of 0.46%.

The optimal dd-cfDNA cutoff point was also determined for the group that underwent biopsy due to suboptimal graft function (SGF) (N = 95). The optimal cutoff for dd-cfDNA was determined to be 0.81%, with a sensitivity of 70.0%, a specificity of 85.9%, and an accuracy of 84.2% for differentiating between active rejection and absence of rejection. The positive predictive value was 36.8%, and the negative predictive value was 96.1%. Most samples (80.0%) had dd-cfDNA levels below the established cutoff point.

## Discussion

Early and accurate detection of kidney graft rejection remains a relevant clinical challenge. Conventional monitoring methods, such as serum creatinine, estimated glomerular filtration rate (eGFR), and biopsy, have limitations related to sensitivity, specificity, and the invasive nature of the procedure^11,12^. In response to this unmet need, dd-cfDNA has emerged as a promising noninvasive biomarker, capable of reflecting tissue injury almost in real time^12^. However, despite extensive international evidence, data on the diagnostic performance of this test in Brazilian KTRs—a population with a high incidence of delayed graft function (DGF) compared with other countries^22^—remain scarce.

This cross-sectional, exploratory, non interventional, single-center study evaluated 492 graft biopsies to investigate the performance of the Prospera dd-cfDNA test as a diagnostic tool for acute rejection in KTRs with a clinical indication for biopsy. In keeping with previous studies and the assay labeling, we initially evaluated the prespecified 1.0% threshold as a clinically meaningful cutoff and then explored data-driven optimal values using ROC curve analysis, which in our cohort suggested a slightly different threshold with comparable overall discrimination.

The diagnostic performance of the dd-cfDNA test in this study was consistent with findings reported in the literature. In the overall cohort, dd-cfDNA values ≥ 1.0% were observed in 14.8% (N = 73) of patients, a proportion very close to the 14.4% (N = 71) of cases undergoing biopsy with histological confirmation of rejection. In addition, dd-cfDNA showed a sensitivity of 50.7% and a specificity of 91.2%, reflecting a satisfactory capacity to identify rejection episodes. The area under the ROC curve was 0.77, indicating moderate performance, in line with previous studies reporting values between 0.71 and 0.88, which varied according to the adopted cutoff and cohort characteristics^15,23,24,25,26,27^. This epidemiologic context has a direct impact on predictive values: even with high specificity (91.2%), the positive predictive value of dd-cfDNA remained modest (48.3%), whereas the negative predictive value was very high (91.6%). In practical terms, a negative dd-cfDNA result at or below 1.0% is reassuring in a setting where the pretest probability of rejection is low and may help clinicians safely defer kidney biopsy in selected patients, whereas a positive result should be interpreted in conjunction with other clinical and laboratory data rather than as a stand-alone confirmatory test. Clinically, these findings suggest that dd-cfDNA is more useful for ruling out rejection (high NPV) than for confirming it, and that positive results should therefore be confirmed by biopsy.

Determining the optimal dd-cfDNA cutoff is essential to optimize its clinical performance as a diagnostic tool for kidney graft rejection. In the present study, the optimal cutoff identified for the overall sample was 0.81%, with a sensitivity of 56.3% and a specificity of 87.6%. This value differs from the cutoff traditionally adopted, which is usually 1.0% for most tests. However, more recent studies have explored lower cutoffs, around 0.5%^15,16,17,18^, reflecting the need for population-specific adjustments and reinforcing the trend that lower values may provide better diagnostic performance in certain clinical contexts.

When stratifying the analysis by clinical indication for biopsy, it was observed that the optimal cutoff for the AGD group was 0.46%, with a sensitivity of 75.4% and a specificity of 68.2%. In the SGF group, the optimal cutoff was 0.81%, with a sensitivity of 70.0% and a specificity of 85.9%. This differentiation underscores the importance of considering the clinical context and graft function at the time of dd-cfDNA assessment, as patients with SGF, often associated with ischemia–reperfusion injury or residual surgical trauma, may present elevated baseline levels of the biomarker in the first months post-transplant^18,28,29^.

Despite the promising performance of dd-cfDNA, it is important to emphasize that no single biomarker replaces kidney biopsy, which continues to provide indispensable information for diagnosis and therapeutic guidance. Thus, the integration of dd-cfDNA with conventional biomarkers has the potential to compose a safer and more individualized diagnostic strategy, being particularly useful for avoiding kidney biopsies in clinical scenarios in which the likelihood of acute rejection is low, given the high negative predictive value and modest positive predictive value observed in our cohort.

This study represents the first evaluation of dd-cfDNA performance in a Brazilian kidney transplant population, characterized by a high incidence of DGF and, consequently, incomplete recovery of graft function. This context highlights the need to generate local evidence on the applicability of dd-cfDNA in this population.

Another relevant aspect is the operational feasibility of using dd-cfDNA in a decentralized model of sample collection, storage, and shipping. The present study demonstrated that this decentralized logistics model is feasible and preserves test integrity, enabling results comparable to international data. Thus, dd-cfDNA proves to be a tool compatible with the logistical reality of the Brazilian healthcare system, potentially expanding the scope of follow-up for transplant recipients. However, the cost of the test represents a significant challenge for its large-scale implementation, both in Brazil and in the context reported by the group of Nissaisorakarn et al.^19^.

This study has some limitations that should be considered when interpreting the results. First, it is a single-center study; however, the analyzed sample is numerically larger than that of most studies available in the literature, which strengthens the robustness of the presented data. Furthermore, the cross-sectional design of the study did not allow for longitudinal monitoring of individual baseline dd-cfDNA levels.

The findings of this study contribute significantly to the advancement of dd-cfDNA use in kidney transplantation, especially in settings with a high incidence of delayed graft function, such as Brazil. In clinical practice, these results support the potential incorporation of dd-cfDNA as a complementary test in the investigation of graft dysfunction, aiding clinical decision-making regarding the need for biopsy.

Multicenter studies with a longitudinal design, involving different centers and population profiles, are essential to expand the external validity of the results. The incorporation of instruments to assess patient quality of life could provide relevant information on the clinical impact of the test and its potential benefits in terms of well-being, particularly considering the reduction in the need for invasive procedures. Additionally, it is imperative to validate and expand the applicability of dd-cfDNA in populations such as Brazil, as well as to establish specific cutoffs tailored to different epidemiological contexts.

The results obtained reinforce its potential for integration into clinical practice, provided that its use is supported by additional studies confirming its performance across different settings. In Brazil, challenges such as cost, implementation, and methodological standardization still need to be addressed to enable its large-scale adoption.

## Data Availability

The datasets generated and/or analyzed during the current study are available from the corresponding author upon reasonable request.
