# Supplementary material for: Diagnostic performance of donor-derived cell-free DNA for acute rejection in kidney allograft biopsies
Source: J Bras Nefrol. 2026 Mar 30;48(3):e20250285. doi: 10.1590/2175-8239-JBN-2025-0285en (PMC13078818; doi:10.1590/2175-8239-JBN-2025-0285en)
Supplement: Translation

# Translation: Desempenho diagnóstico do DNA livre de células derivado do doador para rejeição aguda em biópsias de aloenxerto renal

**DOI:** 10.1590/2175-8239-JBN-2025-0285pt

**Authors:** Mônica Rika Nakamura, Renato Demarchi Foresto, Vitoria Regina da Silva Gomes, Henrique Machado Proença, Lúcio Requião-Moura, José Medina-Pestana, Helio Tedesco-Silva

**Affiliations:** 1 Fundação Oswaldo Ramos, Hospital do Rim, São Paulo, SP, Brasil.; 2 Universidade Federal de São Paulo, Departamento de Medicina, Disciplina de Nefrologia, São Paulo, SP, Brasil.

**Keywords:** Transplante de Rim, Rejeição do Enxerto, DNA Livre de Células Derivado do Doador, Biópsia Renal, Biomarcador Não Invasivo, Diagnóstico

## Abstract

**Objetivo:**

Determinar o desempenho do DNA livre de células derivado do doador (*dd-cfDNA*) como biomarcador não invasivo para o diagnóstico de rejeição aguda em biópsias de transplante renal realizadas por indicação clínica.

**Métodos:**

Estudo transversal, de centro único (maio de 2021 a junho de 2022), que incluiu biópsias realizadas por indicação clínica em receptores de transplante renal com disfunção aguda do enxerto (DAE) ou função insatisfatória do enxerto (FIE). Os níveis de *dd-cfDNA* foram correlacionados com o diagnóstico histológico segundo a classificação de Banff 2022.

**Resultados:**

Entre 492 biópsias, 80,7% foram realizadas por DAE e 19,3% por FIE. A distribuição dos fenótipos histológicos foi: 10,2% (categoria 1), 6,5% (categoria 2), 6,9% (categoria 3), 7,9% (categoria 4), 33,3% (categoria 5) e 35,2% (categoria 6). As medianas correspondentes de *dd-cfDNA* foram 0,25% (IIQ 0,16–0,46), 1,88% (IIQ 0,92–5,11), 0,45% (IIQ 0,26–0,63), 0,51% (IIQ 0,34–1,11), 0,27% (IIQ 0,15–0,45) e 0,38% (IIQ 0,23–0,64). A categoria 2 apresentou mediana de *dd-cfDNA* significativamente mais elevada em comparação com os demais grupos (p < 0,001). A área sob a curva (AUC) para rejeição aguda (categorias 2 e 4) foi 0,77, com sensibilidade de 50,7%, especificidade de 91,2%, valor preditivo positivo de 49,3%, valor preditivo negativo de 91,6% e acurácia de 85,4%. Resultados semelhantes foram observados nas biópsias realizadas por DAE e por FIE. O ponto de corte do *dd-cfDNA* com melhor desempenho diagnóstico para rejeição aguda foi 0,81%, sendo que os valores ideais foram de 0,46% para biópsias por DAE e 0,81% para biópsias por FIE.

**Conclusão:**

Nesta coorte, o *dd-cfDNA* apresentou desempenho diagnóstico moderado para rejeição aguda do enxerto e alto valor preditivo negativo. O melhor ponto de corte para o desempenho diagnóstico do *dd-cfDNA* variou de acordo com o tipo de biópsia realizada por indicação clínica (DAE ou FIE).

## Introdução

A disfunção aguda do enxerto é um dos principais desafios no transplante renal, sendo a rejeição aguda um fator determinante para a perda do enxerto a longo prazo^1,2^. Estudos indicam que a incidência de rejeição aguda varia de 6% a 16% no primeiro ano pós-transplante, dependendo do contexto clínico e das estratégias terapêuticas adotadas^3,4,5,6^.

A suspeita de rejeição aguda frequentemente surge diante do aumento da creatinina sérica, um biomarcador amplamente utilizado devido à sua acessibilidade e baixo custo^7^. Entretanto, sua elevação pode ser tardia e influenciada por fatores extrarrenais, como idade, massa muscular e estado de hidratação do paciente^7^. Além disso, variações nos níveis de creatinina não especificam a causa da disfunção do enxerto, tornando a confirmação diagnóstica por meio da biópsia renal essencial na prática clínica.

A biópsia do enxerto renal é considerada o padrão-ouro para o diagnóstico de rejeição, permitindo a análise histopatológica das lesões e a diferenciação entre rejeição celular e humoral^5^. No entanto, trata-se de um procedimento invasivo, associado a complicações como hematúria macroscópica, hematoma perirrenal e, em casos graves, a demanda por intervenção cirúrgica^8,9,10^. Sendo assim, há necessidade de métodos menos invasivos que permitam uma detecção mais precoce e acurada da rejeição do que as alterações na creatinina sérica, ao mesmo tempo em que complementem, e não substituam, a biópsia renal como padrão diagnóstico.

Nesse contexto, diversos biomarcadores têm sido estudados para fornecer um diagnóstico precoce e não invasivo da rejeição renal^11,12^. O DNA livre de células derivado do doador (*dd-cfDNA*) surgiu como uma ferramenta promissora para o monitoramento da rejeição, permitindo a detecção de episódios de rejeição mediante a quantificação do DNA liberado por células do enxerto na corrente sanguínea do receptor^13^. Evidências sugerem que níveis elevados de *dd-cfDNA* se correlacionam diretamente com eventos de rejeição, reforçando seu potencial como alternativa ou complemento à biópsia renal^14^.

O *dd-cfDNA* apresenta várias vantagens, incluindo a detecção precoce de rejeição e seu caráter não invasivo. Entretanto, seu uso clínico ainda enfrenta desafios, como os elevados custos e a necessidade de valores de corte padronizados para otimizar sua aplicação clínica^15,16,17,18,19^. Ainda assim, a incorporação do *dd-cfDNA* como biomarcador no transplante renal representa um avanço significativo, com potencial de reduzir a necessidade de biópsias renais desnecessárias e contribuir para melhorar os desfechos em longo prazo, ao permitir a detecção e o tratamento mais precoces e precisos da lesão do enxerto.

Apesar das crescentes evidências internacionais, faltam dados sobre o desempenho diagnóstico do *dd-cfDNA* em receptores brasileiros de transplante renal. Portanto, este estudo teve como objetivo avaliar o desempenho diagnóstico do teste Prospera de *dd-cfDNA* como biomarcador não invasivo para rejeição aguda comprovada por biópsia em biópsias de enxerto renal realizadas por indicação clínica em uma coorte brasileira.

## Métodos

Este é um estudo transversal, exploratório, não intervencional e de centro único, desenvolvido para avaliar o desempenho do teste Prospera de *dd-cfDNA* como ferramenta diagnóstica para rejeição aguda em receptores de transplante renal. Entre 17 de maio de 2021 e 6 de junho de 2022, foram triados todos os receptores adultos de transplante renal consecutivos submetidos à biópsia de enxerto renal por indicação clínica.

Foram considerados elegíveis pacientes adultos com 18 anos ou mais, receptores de rins provenientes de doadores vivos ou falecidos, que estavam programados para realizar uma biópsia do enxerto por indicação clínica e concordaram em participar do estudo mediante assinatura do Termo de Consentimento Livre e Esclarecido. Foram excluídos pacientes cuja biópsia foi realizada nos primeiros 15 dias após o transplante, receptores de rim de doador vivo HLA idêntico, indivíduos que haviam recebido previamente outro órgão sólido ou gestantes. Após a confirmação dos critérios de inclusão e exclusão, os pacientes foram convidados a participar do estudo e assinaram o termo de consentimento.

As biópsias foram indicadas por disfunção aguda do enxerto (DAE, definida como elevação da creatinina sérica compatível com possível rejeição aguda ou outra lesão aguda do enxerto) ou por função insatisfatória do enxerto (FIE, definida como recuperação incompleta da função do enxerto no período pós-transplante inicial, não relacionada à recuperação incompleta após episódios tardios de lesão aguda do enxerto), conforme critérios institucionais. Os resultados das biópsias foram analisados por um patologista renal experiente da instituição e classificados de acordo com a Classificação de Banff 2022^20^.

A categoria 1 incluiu biópsias com histologia normal ou apenas alterações mínimas/não específicas. A categoria 2 correspondeu à rejeição mediada por anticorpos. A categoria 3 incluiu alterações limítrofes sugestivas, mas não diagnósticas, de rejeição mediada por células T. Já a categoria 4 compreendeu a rejeição mediada por células T. Na categoria 5, foram incluídos fenótipos de rejeição crônica ou crônica ativa, enquanto a categoria 6 englobou outros diagnósticos não relacionados à rejeição (como doenças glomerulares recorrentes ou *de novo*, fibrose intersticial e atrofia tubular ou lesões não aloimunes). Para as análises de desempenho diagnóstico, biópsias classificadas como categorias 2 ou 4 de Banff foram consideradas rejeição aguda comprovada por biópsia, enquanto aquelas classificadas nas categorias 1, 3, 5 e 6 foram agrupadas como fenótipos sem rejeição.

Para a avaliação do *dd-cfDNA*, duas amostras de sangue periférico (10 mL cada) foram coletadas em tubos com EDTA imediatamente antes do procedimento de biópsia. As amostras de plasma foram processadas conforme as instruções do fabricante, centrifugadas, armazenadas a –80 °C e posteriormente enviadas, sob controle de temperatura, ao laboratório da Natera (San Carlos, CA, EUA) para análise pelo ensaio Prospera. Os níveis de *dd-cfDNA* foram quantificados por meio de sequenciamento de nova geração e expressos como a porcentagem de fragmentos derivados do doador em relação ao total de DNA livre no plasma. Para a análise primária, e em consonância com estudos prévios e com a brochura do teste Prospera, um valor de *dd-cfDNA* ≥ 1,0% foi pré-especificado como resultado positivo para a detecção de rejeição aguda comprovada por biópsia. Os resultados do teste não foram utilizados para orientar decisões clínicas, não interferindo na conduta médica.

Dados clínicos e laboratoriais foram extraídos dos prontuários eletrônicos institucionais utilizando um banco de dados padronizado. Variáveis contínuas foram apresentadas como medianas e intervalos interquartílicos (IIQ), e variáveis categóricas, como números absolutos e percentuais. A distribuição dos valores de *dd-cfDNA* entre as categorias histológicas de Banff foi explorada utilizando testes não paramétricos para amostras independentes.

Primeiramente, avaliamos o desempenho diagnóstico do ponto de corte pré-especificado de 1,0% para a detecção de rejeição aguda comprovada por biópsia, calculando sensibilidade, especificidade, valor preditivo positivo, valor preditivo negativo e acurácia global, com seus respectivos intervalos de confiança de 95%. Em seguida, curvas ROC (*Receiver Operating Characteristic*) foram construídas, e a área sob a curva (AUC) foi estimada. Os pontos de corte ótimos de *dd-cfDNA* para a coorte total e para os subgrupos de biópsias realizadas por DAE e por FIE foram determinados pelo índice de *Youden*, sem fixação prévia do limiar de 1,0%.

Todas as análises estatísticas foram realizadas utilizando o software *Statistical Package for the Social Sciences* (SPSS) versão 29 (IBM Corp., 2022)^21^. Um valor de p < 0,05 foi considerado estatisticamente significativo, com nível de confiança de 95%. Os dados clínicos foram extraídos dos prontuários eletrônicos da instituição, inseridos na plataforma REDCap e posteriormente exportados para análise estatística. O protocolo do estudo e o termo de consentimento foram submetidos e aprovados pelo Comitê de Ética em Pesquisa (número de aprovação: 6.566.447). Todos os procedimentos do estudo foram realizados somente após a aprovação ética.

## Resultados

A população do estudo consistiu em 492 biópsias provenientes de 446 receptores de transplante renal, predominantemente do sexo masculino (65%) e de raça branca (63,4%), com mediana de idade de 44 anos (IIQ: 34–55 anos). Entre os receptores, 48,6% apresentavam doença renal crônica de etiologia indeterminada. Além disso, 95,1% eram receptores de primeiro transplante renal e 96,1% haviam realizado hemodiálise previamente. Quanto aos doadores, a mediana de idade foi de 48 anos (IIQ: 38–57 anos), 53,7% eram do sexo masculino e 54,3%, brancos. Houve predominância de doadores falecidos (77,2%), e 23,9% apresentavam *Kidney Donor Profile Index* (KDPI) > 85%. Em relação às características do transplante, qualquer positividade de anticorpos reativos contra painel (PRA) foi observada em 23,2% dos pacientes para classe I e 8,7% para classe II. O número mediano de incompatibilidades ABDR foi 2,5. O tempo médio de isquemia fria foi de 19,0 horas, e 141 pacientes (28,7%) apresentaram função tardia do enxerto (FTE). A principal indicação da biópsia foi disfunção aguda do enxerto (DAE), observada em 80,7% dos casos. Os demais 19,3% foram submetidos ao procedimento devido à função insatisfatória do enxerto (FIE). O tempo mediano entre o transplante e a biópsia foi de 17,5 meses (IIQ: 3,0–52,2 meses) (Figura 1, Tabela 1).

**Figura 1:** Disposição das amostras do estudo.

**Tabela 1:** Demografia da população do estudo

Variável	Total (N = 492)	DAE (N = 397)	FIE (N = 95)
Idade do receptor, anos (IIQ)	44 (34,0–55,0)	45 (34,0–55,0)	43 (34,5–55,0)
Sexo do receptor, masculino, N (%)	318 (65)	244 (61,5)	53 (64)
Raça do receptor, N (%)			
Branca	312 (63,4)	258 (65,0)	54 (56,8)
Negra/Parda	179 (36,4)	138 (34,8)	41 (43,2)
Outras	1 (0,2)	1 (0,3)	0 (0)
Causa da doença renal crônica, N (%)			
Indeterminada	239 (48,6)	193 (48,6)	46 (48,4)
Glomerulonefrite	94 (19,0)	81 (20,4)	13 (13,7)
Diabetes mellitus	51 (10,4)	44 (11,1)	7 (7,4)
Outras	108 (22,0)	79 (19,9)	29 (30,5)
Tempo de diálise, meses (IIQ)	30 (16,0–59,0)	30 (16,0–60,0)	30 (16,0–58,0)
Tipo de tratamento, hemodiálise, N (%)	473 (96,1)	380 (95,7)	93 (97,9)
Retransplante, N (%)	24 (4,9)	19 (4,8)	5 (5,3)
Idade do doador, anos (IIQ)	48 (38,0–57,0)	47 (38,0–56,0)	52 (42,0–58,5)
Sexo do doador, masculino, N (%)	264 (53,7)	211 (53,1)	53 (55,8)
Raça do doador, N (%)			
Branca	267 (54,3)	217 (54,7)	50 (52,6)
Negra/Parda	221 (44,9)	176 (44,3)	45 (47,4)
Outras	4 (0,8)	4 (1,0)	0 (0)
Tipo de doador, falecido, N (%)	380 (77,2)	296 (74,6)	84 (88,4)
Doadores falecidos			
KDPI, % (IIQ)	65 (44,0–84,3)	64 (44,0–82,0)	67 (48,0–88,0)
KDPI > 85%, N (%)	91 (23,9)	67 (23,0)	24 (29,0)
PRA Classe I > 0%, N (%)	114 (23,2)	98 (24,7)	16 (16,8)
PRA Classe II > 0%, N (%)	43 (8,7)	39 (9,8)	4 (4,2)
Tempo de isquemia fria, horas (IIQ)	19,0 (10,0–25,0)	19,0 (2,0–24,0)	23,0 (15,0–30,0)
Função tardia do enxerto, N (%)	141 (28,7)	107 (27,0)	34 (35,8)
Indução, globulina anti-timócito, N (%)	491 (99,8)	397 (100)	94 (98,9)
Imunossupressão, N (%)			
Tacrolimo + Micofenolato	256 (52,0)	200 (50,4)	56 (58,9)
Tacrolimo + Azatioprina	152 (30,9)	123 (31,0)	29 (30,5)
Tacrolimo + inibidor de mTOR	68 (13,8)	59 (14,9)	9 (9,5)
Outros	16 (3,3)	15 (3,8)	1 (1,1)
Tempo pós transplante, meses (IIQ)	17,5 (3,0–52,2)	31 (8,2–63,2)	1,4 (1,2–2,0)
Tratamento pós biópsia, sim, N (%)	106 (21,5)	92 (22,9)	14 (14,7)
Metilprednisolona	90 (84,9)	78 (84,8)	12 (85,7)
Globulina anti-timócito	7 (6,6)	5 (5,4)	2 (14,3)
Imunoglobulina	3 (2,8)	3 (3,3)	0 (0)
Metilprednisolona + imunoglobulina	4 (3,8)	4 (4,3)	0 (0)
Antibiótico	2 (1,9)	2 (2,2)	0 (0)

Abreviaturas – N: Número; DAE: Disfunção Aguda do Enxerto; FIE: Função Insatisfatória do Enxerto; IIQ: Intervalo Interquartil; KDPI: *Kidney Donor Profile Index*; PRA: Anticorpo Reativo ao Painel; HLA: Antígeno Leucocitário Humano; mTOR: inibidor da via *mammalian target of rapamycin*. Nota – valores expressos como mediana (1º IIQ; 3º IIQ) e valores absolutos (%).

Foram realizados 492 testes de *dd-cfDNA*, com média de 0,81%, variando de 0,01% a 21,02%. A mediana foi de 0,35% (IIQ: 0,18–0,63%). A maior parte da população, totalizando 419 casos (85,2%), apresentou valores de *dd-cfDNA* inferiores a 1%.

A Figura 2, que apresenta um *boxplot* das porcentagens de *dd-cfDNA* de acordo com a classificação de Banff 2022, demonstra maior dispersão e valores mais elevados na categoria 2 quando comparada às demais categorias (p < 0,001). Rejeição aguda comprovada por biópsia foi identificada em 71 biópsias, correspondendo a 14,4% de todas as biópsias por indicação clínica da coorte. Destas, 32 foram classificadas como rejeição mediada por anticorpos (categoria 2 de Banff) e 39 como rejeição mediada por células T (categoria 4). A distribuição dos fenótipos histológicos foi: 10,2% (N = 50) na categoria 1, 6,5% (N = 32) na categoria 2, 6,9% (N = 34) na categoria 3, 7,9% (N = 39) na categoria 4, 33,3% (N = 164) na categoria 5 e 35,2% (N = 173) na categoria 6. As respectivas medianas de *dd-cfDNA* foram 0,25% (IIQ 0,16–0,46), 1,88% (IIQ 0,92–5,11), 0,45% (IIQ 0,26–0,63), 0,51% (IIQ 0,34–1,11), 0,27% (IIQ 0,15–0,45) e 0,38% (IIQ 0,23–0,64).

**Figura 2:** *Boxplot* mostrando o teste de *dd-cfDNA* e a classificação de Banff 2022.

Utilizando o ponto de corte pré-especificado de 1,0% para definir teste positivo de *dd-cfDNA*, o ensaio apresentou sensibilidade de 50,7% e especificidade de 91,2% para a detecção de rejeição aguda comprovada por biópsia, com acurácia global de 85,4%. A área sob a curva (AUC) foi 0,77, indicando boa capacidade do teste em discriminar pacientes com e sem rejeição. O valor preditivo positivo (VPP) foi 49,3% e o valor preditivo negativo (VPN) atingiu 91,6% (Figura 3).

**Figura 3:** Análise do desempenho do teste de *dd-cfDNA* na população total.

O ponto de corte ideal para o *dd-cfDNA* na detecção de rejeição aguda foi determinado para o grupo total (N = 492) utilizando o Índice de *Youden*. A análise revelou um ponto de corte de 0,81%, com sensibilidade de 56,3%, especificidade de 87,6% e acurácia de 83,1%. O VPP foi de 43,5%, e o VPN de 92,3%. A maioria das amostras (81,3%) apresentou níveis de *dd-cfDNA* abaixo desse ponto de corte. Para o grupo submetido à biópsia devido à disfunção aguda do enxerto (DAE) (N = 397), o ponto de corte ideal de *dd-cfDNA* foi 0,46%, com sensibilidade de 75,4%, especificidade de 68,2% e acurácia de 69,3%. O VPP foi 30,1% e o VPN, 93,9%. Nesta população, 61,5% das amostras apresentaram valores de *dd-cfDNA* abaixo do ponto de corte de 0,46%.

Para o grupo submetido à biópsia devido à função insatisfatória do enxerto (FIE) (N = 95), o ponto de corte ideal de *dd-cfDNA* também foi determinado. O valor ótimo encontrado foi 0,81%, com sensibilidade de 70,0%, especificidade de 85,9% e acurácia de 84,2%. Nesse cenário, o VPP foi 36,8% e o VPN, 96,1%. A maioria das amostras (80,0%) apresentou níveis de *dd-cfDNA* abaixo desse ponto de corte.

## Discussão

A detecção precoce e precisa da rejeição do enxerto renal permanece um importante desafio clínico. Métodos convencionais de monitoramento, como a creatinina sérica, a taxa de filtração glomerular estimada (TFGe) e a biópsia, apresentam limitações relacionadas à sensibilidade, à especificidade e ao caráter invasivo do procedimento^11,12^. Diante dessa necessidade não atendida, o *dd-cfDNA* emergiu como um biomarcador não invasivo promissor, capaz de indicar lesão tecidual praticamente em tempo real^12^. No entanto, apesar das numerosas evidências internacionais, ainda há escassez de dados sobre o desempenho diagnóstico desse teste em receptores de transplante renal brasileiros, uma população com alta incidência de função insatisfatória do enxerto (FIE) e de função tardia do enxerto (FTE) em comparação a outros países^22^.

Este estudo transversal, exploratório, não intervencional e de centro único avaliou 492 biópsias renais para investigar o desempenho do teste Prospera de *dd-cfDNA* como ferramenta diagnóstica de rejeição aguda em receptores com indicação clínica para biópsia. Em consonância com estudos prévios e com a brochura do ensaio, inicialmente avaliamos o ponto de corte pré-especificado de 1,0% como limiar clinicamente relevante, e posteriormente exploramos valores ideais orientados por dados por meio da análise da curva ROC, que, em nossa coorte, sugeriu um limiar ligeiramente diferente, mas com discriminação global semelhante.

O desempenho diagnóstico do *dd-cfDNA* observado neste estudo foi consistente com achados da literatura. Na coorte geral, valores de *dd-cfDNA* ≥ 1,0% foram observados em 14,8% (N = 73) dos pacientes, proporção muito próxima dos 14,4% (N = 71) de casos com confirmação histológica de rejeição. Além disso, o *dd-cfDNA* apresentou sensibilidade de 50,7% e especificidade de 91,2%, refletindo uma capacidade satisfatória de identificar episódios de rejeição. A área sob a curva ROC foi de 0,77, indicando desempenho moderado, alinhado a estudos prévios que reportam valores entre 0,71 e 0,88, variando de acordo com o ponto de corte adotado e as características da coorte^15,23,24,25,26,27^. Esse contexto epidemiológico impacta diretamente os valores preditivos: mesmo com alta especificidade (91,2%), o valor preditivo positivo do *dd-cfDNA* permanece modesto (48,3%), enquanto o valor preditivo negativo atinge patamar muito elevado (91,6%). Em outras palavras, um resultado negativo de *dd-cfDNA* ≤ 1,0% é tranquilizador em um cenário de baixa probabilidade pré-teste de rejeição e pode auxiliar o clínico a postergar a realização de biópsia em casos selecionados, enquanto um resultado positivo deve ser interpretado em conjunto com outros dados clínicos e laboratoriais, não como teste confirmatório isolado. Clinicamente, esses achados sugerem que o *dd-cfDNA* é mais útil para excluir rejeição (alto VPN) do que para confirmá-la, justificando a necessidade de biópsia diante de resultados positivos.

A definição de um ponto de corte ideal para o *dd-cfDNA* é fundamental para otimizar seu desempenho clínico como ferramenta diagnóstica na rejeição do enxerto renal. No presente estudo, o ponto de corte ideal identificado para a amostra total foi de 0,81%, com sensibilidade de 56,3% e especificidade de 87,6%. Esse valor difere do ponto de corte tradicionalmente adotado, geralmente em torno de 1,0%, mas estudos recentes têm proposto valores mais baixos, por volta de 0,5%^15,16,17,18^, refletindo a necessidade de ajustes para populações específicas e confirmando a tendência de que valores menores podem oferecer melhor desempenho diagnóstico em determinados contextos clínicos.

Ao estratificar a análise pela indicação clínica de biópsia, observou-se que o ponto de corte ideal para o grupo com disfunção aguda do enxerto (DAE) foi de 0,46%, com sensibilidade de 75,4% e especificidade de 68,2%. No grupo com função insatisfatória do enxerto (FIE), o ponto de corte ideal foi de 0,81%, com sensibilidade de 70,0% e especificidade de 85,9%. Essa diferenciação destaca a importância de considerar o contexto clínico e o *status* funcional do enxerto no momento da avaliação do *dd-cfDNA*, uma vez que pacientes com FIE, frequentemente associada à lesão de isquemia-reperfusão ou a trauma cirúrgico residual, podem apresentar valores basais elevados do biomarcador nos primeiros meses pós-transplante^18,28,29^.

Apesar do desempenho promissor do *dd-cfDNA*, é importante enfatizar que nenhum biomarcador isolado substitui a biópsia renal, que continua fornecendo informações indispensáveis tanto para o diagnóstico quanto para a orientação terapêutica. Entretanto, a integração do *dd-cfDNA* com biomarcadores convencionais tem potencial para compor uma estratégia diagnóstica mais segura e individualizada, sendo particularmente útil para evitar a realização de biópsias renais em cenários clínicos nos quais a probabilidade de rejeição é baixa, situação sustentada pelo alto valor preditivo negativo e pelo modesto valor preditivo positivo observados em nossa coorte.

Este estudo representa a primeira avaliação do desempenho do *dd-cfDNA* em uma população brasileira de receptores de transplante renal, caracterizada pela alta incidência de FTE e, consequentemente, pela recuperação incompleta da função do enxerto. Esse contexto reforça a necessidade de gerar evidências locais sobre a aplicabilidade do *dd-cfDNA* nessa população específica.

Outro aspecto relevante é a viabilidade operacional do uso do *dd-cfDNA* em um modelo descentralizado de coleta, armazenamento e envio de amostras. O presente estudo demonstrou que essa logística é factível e preserva a integridade do teste, possibilitando resultados comparáveis aos observados em estudos internacionais. Assim, o *dd-cfDNA* mostra-se compatível com a realidade logística do sistema de saúde brasileiro, ampliando potencialmente o monitoramento de receptores de transplante. No entanto, o custo do teste constitui um desafio significativo para sua implementação em larga escala, tanto no Brasil quanto no cenário descrito pelo grupo de Nissaisorakarn et al.^19^.

Este estudo apresenta algumas limitações que devem ser consideradas na interpretação dos resultados. Primeiramente, trata-se de um estudo de centro único; entretanto, a amostra analisada é numericamente maior do que a de grande parte dos estudos disponíveis, conferindo robustez aos dados apresentados. Além disso, o desenho transversal não permitiu o acompanhamento longitudinal dos valores basais individuais de *dd-cfDNA*.

Os achados deste estudo contribuem de forma significativa para o avanço do uso do *dd-cfDNA* no transplante renal, especialmente em contextos com alta incidência de FTE, como no Brasil. Na prática clínica, esses resultados sustentam a possibilidade de incorporar o *dd-cfDNA* como exame complementar na investigação de disfunção do enxerto, auxiliando o clínico na decisão sobre a necessidade de realizar biópsia.

Estudos multicêntricos, longitudinais e envolvendo diferentes perfis populacionais são fundamentais para ampliar a validade externa dos resultados. A incorporação de instrumentos de avaliação de qualidade de vida poderia fornecer informações relevantes sobre o impacto clínico do teste e seu potencial benefício em termos de bem-estar, considerando a redução de procedimentos invasivos. Adicionalmente, é essencial validar e expandir a aplicabilidade do *dd-cfDNA* em populações como a brasileira, bem como estabelecer pontos de corte específicos para diferentes contextos epidemiológicos.

Os resultados obtidos reforçam o potencial do *dd-cfDNA* para integração à prática clínica, desde que acompanhado por estudos adicionais que confirmem seu desempenho em diferentes realidades. No Brasil, desafios como o custo, a implementação e a padronização metodológica ainda precisam ser enfrentados para possibilitar sua adoção em larga escala.

## Data Availability

Os conjuntos de dados gerados e/ou analisados durante o presente estudo estão disponíveis junto ao autor correspondente, mediante solicitação razoável.
